# Urine podocyte mRNA loss in preterm infants and related perinatal risk factors

**DOI:** 10.1007/s00467-022-05663-6

**Published:** 2022-06-27

**Authors:** Qi Gao, Congchao Lu, Xiuying Tian, Jun Zheng, Fangrui Ding

**Affiliations:** 1 Department of Neonatology, Tianjin Central Hospital of Obstetrics and Gynecology, No. 156 Nan Kai San Ma Lu, Tianjin, 300000 China; 2 Tianjin Key Laboratory of Human Development and Reproductive Regulation, Tianjin, 300000 China; 3 Department of Neonatology, Nankai University Maternity Hospital, Tianjin, 300000 China; 4 School of Public Health, Tianjin Medical University, Tianjin, 300000 China

**Keywords:** Preterm infant, Podocyte, Chronic kidney disease

## Abstract

**Background:**

Preterm birth has been identified as a risk factor for development of long-term chronic kidney disease. Podocyte loss has been reported to contribute to this process in preterm animal models. However, details about podocyte loss in preterm infants and related perinatal risk factors have not been well clarified.

**Methods:**

Forty full-term infants and 106 preterm infants were enrolled. Urine samples were collected from full-term infants within 4–7 days of birth and preterm infants at 37–40 weeks of corrected age. Levels of urine podocin mRNA, urine protein (UP), and urine microalbumin (UMA) were measured, and the relationship between these markers was evaluated. Clinical information in these infants was collected, and potential correlates that may lead to increased podocyte loss during the perinatal period were identified using linear regression analysis.

**Results:**

Urine podocyte loss indicated by the urine podocin mRNA to creatinine ratio (UpodCR) was higher in preterm infants than in full-term infants. UpodCR was correlated with the levels of UP and UMA. Multiple linear regression analysis also showed that lower gestational age (GA) at birth and small for gestational age (SGA) were high risk factors for urine podocyte loss.

**Conclusions:**

Increasing urine podocyte loss was identified in preterm infants. Moreover, perinatal factors were associated with podocyte loss and may be a potential direction for comprehensive research and intervention in this field.

**Graphical Abstract:**

A higher resolution version of the Graphical abstract is available as Supplementary information

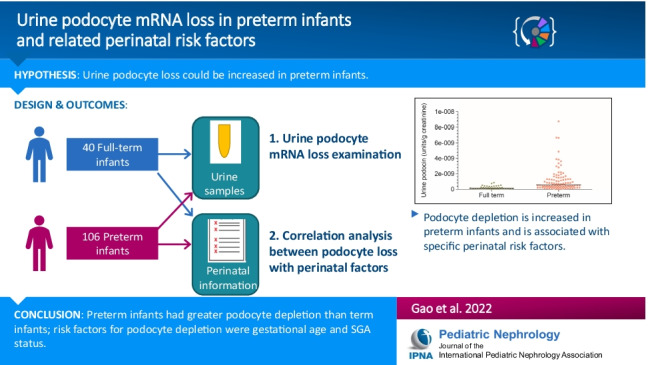

**Supplementary Information:**

The online version contains supplementary material available at 10.1007/s00467-022-05663-6.

## Introduction

The developmental origins of health and disease theory indicate that adverse factors present in early life could explain the increasing burden of each system’s diseases in children and adults [1–3]. Prematurity is one of the adverse factors during the perinatal period and a proven risk factor for disorders affecting various body systems such as the neurological, respiratory, metabolic, cardiovascular, and kidney systems [4–9]. In the field of kidney diseases, many clinical studies have shown that preterm birth is a risk factor for chronic kidney disease (CKD) [4–6]. In a national cohort study conducted in 2019, Crump et al. reported that preterm birth was a strong risk factor for CKD development from childhood into mid-adulthood [5]. The study included more than four million people, and gestational age (GA) at birth was inversely associated with CKD risk. Compared with full-term birth, preterm and extremely preterm births were associated with nearly twofold and threefold higher risks of CKD, respectively [5]. In addition to the results of clinical studies, others have hypothesized that the underlying mechanism of the higher risk of later CKD development due to preterm birth is caused by the lower number of nephrons in preterm infants [10–13]. In humans, nephron development begins at the 9th gestational week, rapidly proliferates in the last trimester, and ceases in approximately the 36th week [14–17]. The GA of premature infants ranges from 21 to 37 weeks, corresponding to the last trimester, which indicates that prematurity interrupts the normal physiological development of the kidneys [14, 16, 18].

In addition to the hypothesis of lower nephron numbers, our recent study [19] also put forward the involvement of the podocyte depletion hypothesis [20–23]. In that study, a preterm rat model was established by delivery 2 days early. Fewer podocytes in glomeruli and higher levels of podocyte excretion into urine were detected in preterm rats. In addition, we followed up these preterm rats until age 12 months, which corresponded to middle adulthood in humans. Persistent podocyte depletion and gradually accelerated podocyte loss were detected in preterm rats, which indicated a high risk of development of CKD. Although that study identified the role of podocyte loss caused by preterm birth in long-term CKD in animal studies, there are many differences between preterm rats and preterm infants because preterm infants experience a much more complex disease process, such as asphyxia, infection, oxygen therapy, drug exposure, and various maternal factors. Podocyte loss in preterm infants has rarely been reported [19, 24]. In addition, it is also unclear which perinatal factors affect podocyte loss. Thus, in the present study, a large number of preterm infants and related clinical and perinatal information were included. Urinary podocyte loss and urine protein (UP) and urine microalbumin (UMA) were measured and then analyzed to comprehensively clarify the mechanism of podocyte loss in premature infants, and perinatal risk factors related to podocyte loss were also analyzed.

## Methods

This study was approved by the Tianjin Central Hospital of Gynecology and Obstetrics Institutional Review Board (No. 2020KY049). All study protocols adhered to the Declaration of Helsinki, and written informed consent was obtained from all parents of the participants before their inclusion in this study.

### Participants

Infants born from 1 July 2019 to 30 November 2019 were included in this study. Infants with known kidney diseases, chromosomal or congenital anomalies, or acute infection were excluded. After obtaining consent from their parents, 106 preterm infants and 40 full-term infants were enrolled. Detailed perinatal information was obtained from medical records. In addition, preterm infants were divided into three groups for further comparison, including ≤ 30 weeks (*n* = 41), 31–34 weeks (*n* = 38), and 35–36 weeks (*n* = 27).

### Urine sample collection

In the full-term group, to eliminate the maternal creatinine effect, urine samples were collected 4–7 days after birth. In this group, routine urine tests were performed in all urine samples. Samples with positive urine protein, occult blood, or white blood cell results were excluded from further processing. In the preterm group, urine samples were collected at 37–41 corrected gestational weeks to match the age of the full-term group. For each single infant, collection bags were applied and removed every 3 h and then stored at 4 °C. When the total urine volume for each participant reached > 30 mL, all of the samples collected from each single participant were sent to the laboratory and pooled together for further processing.

### Human urine sample processing and examination

The methods used were as previously described [25–27] and are detailed as follows. After the urine samples were sent to the laboratory, the samples were centrifuged at 4 °C for 15 min at 4000 rpm. Before the supernatant was discarded, 2 mL of urine supernatant was stored for creatinine and protein testing. UP and UMA levels were detected by turbidimetric tests (Mindray BS-480, Horiba ABX SA, France) using urine supernatant. After removing the remaining supernatant, the urine pellet was washed by DEPC-PBS twice and re-centrifuged at 12,000 rpm for 5 min at 4 °C. The supernatant was removed, and RLT buffer from the RNeasy Mini Kit (Qiagen, Germany, Catalog No. 74106) was added, and the sample was stored at − 80 °C or processed immediately for RNA extraction. RNA was extracted following the manual instructions for the RNeasy Mini Kit.

After RNA extraction, quantitation of podocin mRNA was detected by TaqMan probes for human NPHS2 (podocin) (Catalog No. Hs00922492_m1) by using the 7500 Fast Real-Time PCR System (Applied Biosystems, MA, USA). Standard curves were constructed for each assay using serially diluted cDNA standards. Podocin cDNA of known sequence and concentration was used as a standard for each assay so that the data could be calculated on a molar basis for each probe. Finally, urine podocin mRNA was corrected per gram of creatinine and expressed as urine podocin mRNA to creatinine ratio (UpodCR).

### Statistical analysis

The median and the 25th and 75th percentiles were used to describe the measurement data among the groups. The differences between term and preterm infants were analyzed using the Mann–Whitney *U* test. Comparisons among multiple groups were analyzed by using the Kruskal–Wallis H-rank sum test followed by the Bonferroni post hoc adjustment. Spearman rank correlation was used to analyze the correlation among indicators since the data were skewed. Univariate and multivariate linear regression analyses were used to analyze the risk factors that may lead to increased podocyte loss during the perinatal period. For multivariate linear regression analysis, variables included were those with *p*-value < 0.2 identified in the univariate linear regression analysis. The test level was 0.05. IBM SPSS Statistics 26.0 for Windows (IBM Corp., Armonk, NY, USA) was used for this study, with a test level *α* = 0.05, and analyses were conducted in 2021.

## Results

### Basic clinical information of full-term and preterm infants

In this study, 146 neonates were enrolled, including 40 full-term infants (≥ 37 weeks) and 106 premature infants (< 37 weeks). The baseline clinical information is shown in Table 1. Given the wide range in GA in the preterm group, infants were divided into three groups according to GA, and the detailed baseline information for each group is also given in Table 1.

**Table 1:** Characteristics of the full-term and preterm infants

Variable	Full-term infant (≥ 37 W) *n* = 40	All preterm infant (< 37 W) *n* = 106	Preterm infant		
			35–36 W *n* = 27	31–34 W *n* = 38	≤ 30 W *n* = 41
Gestation (weeks)	39.21 ± 1.17	32.16 ± 3.22	35.76 ± 0.52	33.67 ± 0.91	28.39 ± 0.91
Male (no./percent)	18/45.0%	62/58.5%	19/70.4%	33/86.8%	10/24.4%
Vaginal delivery (no./percent)	27/67.5%	45/42.5%	9/33.3%	13/34.2%	23/56.1%
Single birth (no./percent)	40/100.0%	56/52.8%	15/55.6%	19/50.0%	22/53.7%
Birth height (cm)	50.02 ± 0.95	41.71 ± 4.30	45.37 ± 2.94	43.71 ± 2.59	37.44 ± 2.30
Birth weight (g)	3283.00 ± 330.39	1704.25 ± 542.25	2191.48 ± 530.40	1901.32 ± 333.95	1200.73 ± 169.74
Small for gestational age (no./percent)	0/0.0%	25/23.6%	14/51.9%	9/23.7%	2/4.9%
Apgar score (at 1 min)	9.85 ± 0.53	8.30 ± 1.38	9.37 ± 0.84	8.92 ± 0.75	7.02 ± 1.11
Maternal age (years)	30.93 ± 4.42	30.82 ± 4.31	30.19 ± 3.91	30.18 ± 5.20	31.83 ± 3.46
Maternal hypertension (no./percent)	4/10.0%	18/17.0%	6/22.2%	8/21.1%	4/9.8%
Maternal diabetes mellitus (no./percent)	7/17.5%	22/20.8%	7/25.9%	7/18.4%	8/19.5%
Preeclampsia (no./percent)	3/7.5%	12/11.3%	5/18.5%	4/10.5%	3/7.3%
Neonatal intracranial hemorrhage (no./percent)	2/5.0%	12/11.3%	3/11.1%	4/10.5%	5/12.2%
Retinopathy of prematurity (no./percent)	0/0.0%	10/9.4%	0/0.0%	0/0%	10/24.4%
Neonatal infection (no./percent)	0/0.0%	51/48.1%	4/14.8%	10/26.3%	37/90.2%
Administration of ibuprofen (no./percent)	0/0.0%	22/20.8%	0/0.0%	0/0%	22/53.7%

*no.*: number

### Podocyte loss in preterm infants

**Fig. 1:**
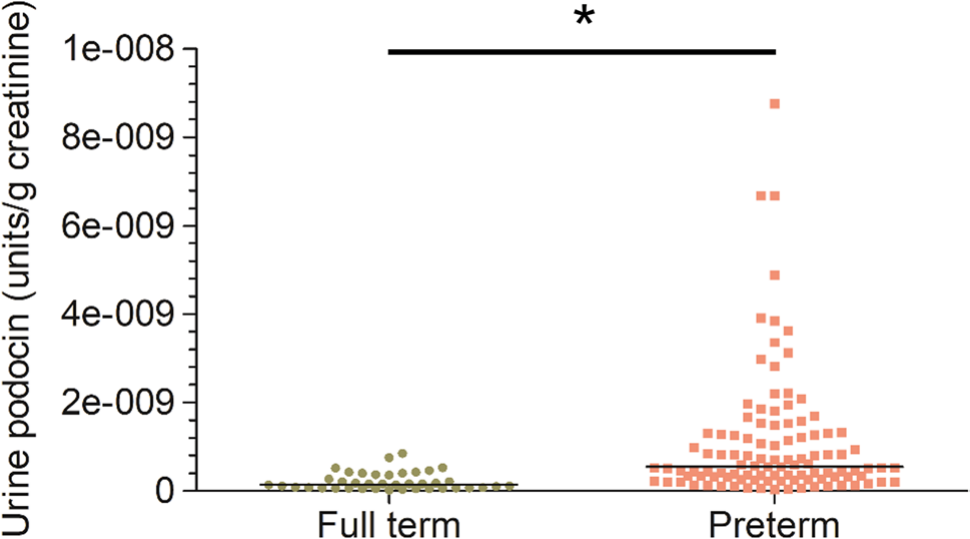
High levels of urine podocyte mRNA in preterm infants. Measurements of podocyte mRNA depletion in the urine pellet were expressed as the urine podocin mRNA to creatinine ratio (analogous to the urine protein to creatinine ratio). Urine podocin mRNA levels from full-term (*n* = 40) and preterm (*n* = 106) infants were compared. Preterm infants had a 5.05-fold increase in podocin mRNA in the urine pellet (full-term vs. preterm: 2.168E-10 units vs. 1.099E-09 units) (Mann–Whitney rank sum tests were used to compare urine podocin mRNA levels between preterm and full-term infants, *p < 0.05)

Urine pellet podocin mRNA was examined to indicate podocyte loss and adjusted to urine creatinine. As shown in Fig. 1, UpodCR was higher in the preterm group than in the full-term group (*Z* =  − 6.073, *p* < 0.001).

### Podocyte loss in relation to GA

**Fig. 2:**
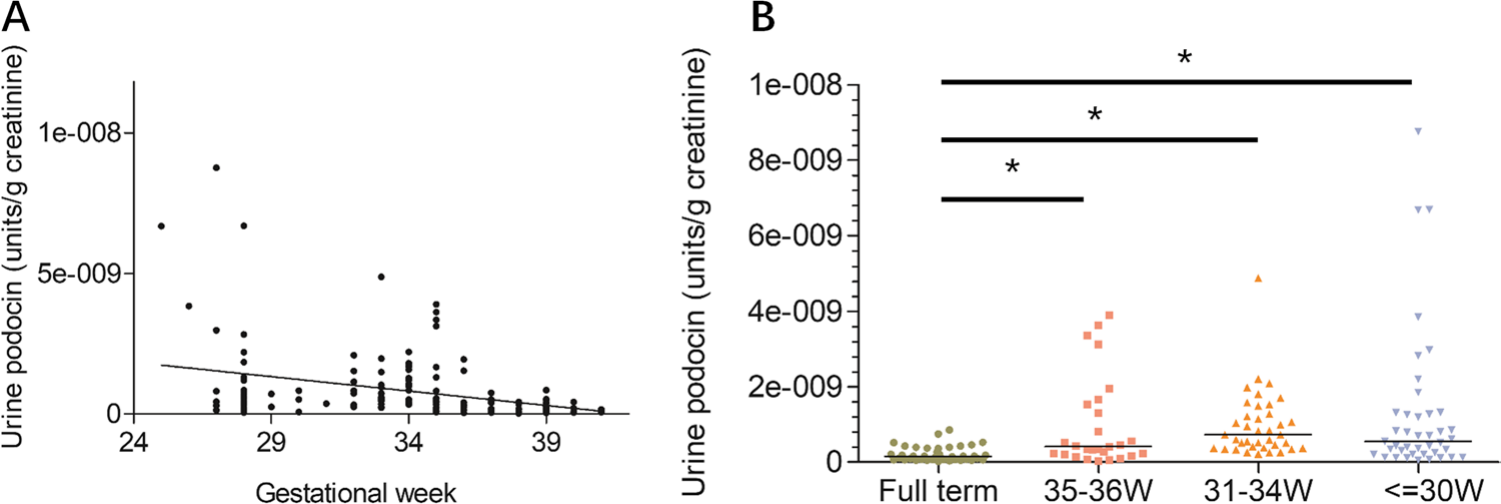
Urine podocyte mRNA levels in relation to GA. (**A**) The Spearman rank correlation test was performed to examine the correlation between urine podocyte mRNA and GA, and a negative correlation was found between GA at birth and urine podocyte loss (*ρ* =  − 0.437, *p* < 0.001). (**B**) Preterm infants were divided into three groups according to GA: 35–36 weeks group (*n* = 27), 31–34 weeks group (*n* = 38), and ≤ 30 weeks group (*n* = 41). These preterm groups had statistically increased urine podocin mRNA excretion in the urine pellet, but no difference was found among all preterm groups (Mann–Whitney rank sum test, *p < 0.05)

The relationship between podocyte loss in preterm infants and GA was examined. As shown in Fig. 2A, podocyte loss indicated by UpodCR was negatively correlated to GA (*ρ* =  − 0.437, *p* < 0.001). As shown in Fig. 2B, podocyte loss was increased in all three preterm groups even in the near-term group (35–36 weeks), and no significant differences were found between any of the preterm groups.

### UP and UMA in preterm infants

**Fig. 3:**
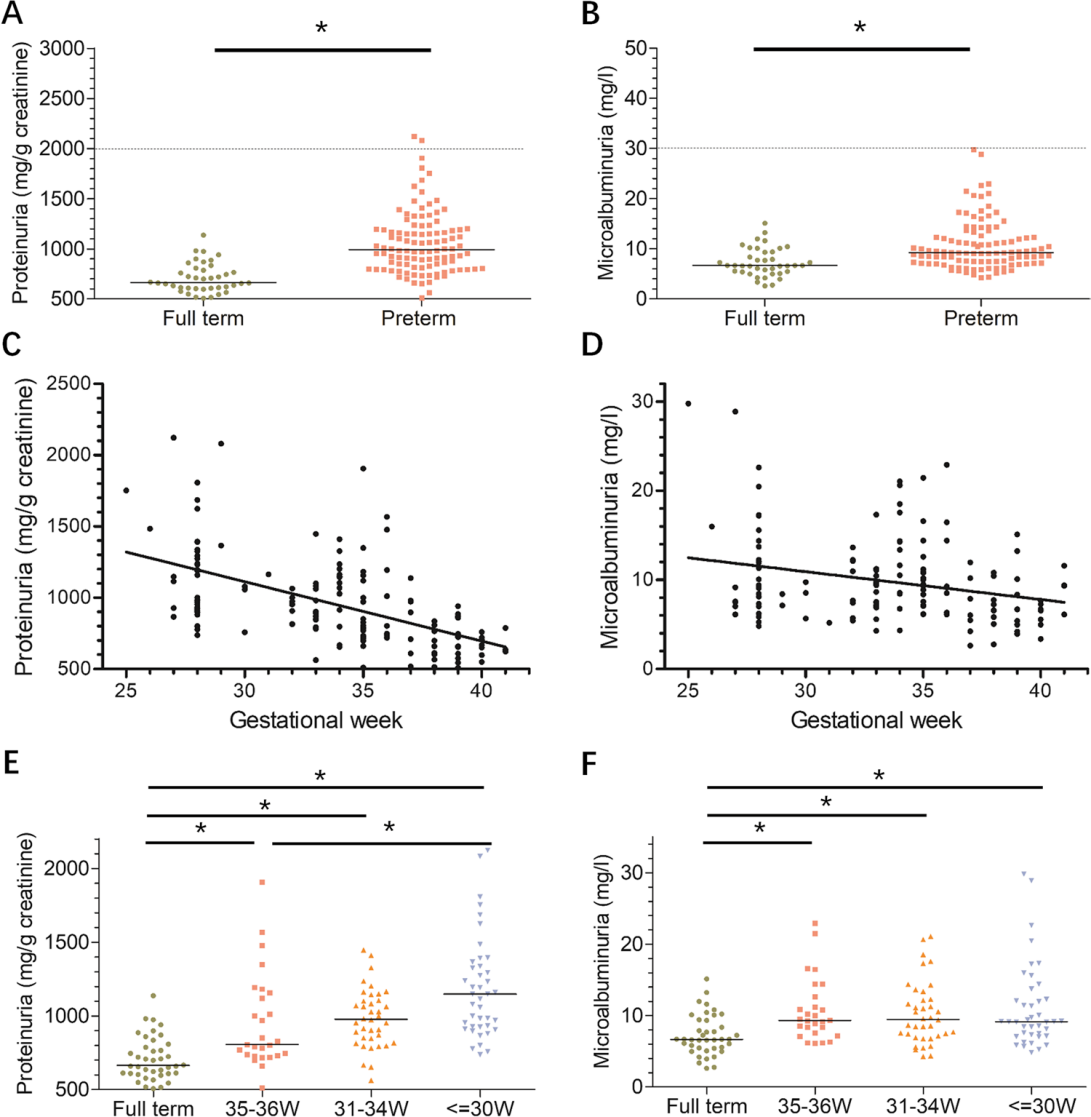
Worsening proteinuria and microalbuminuria were detected in preterm infants and negatively correlated with GA. (**A**, **B**) UP and UMA were examined and compared between full-term infants (*n* = 40) and preterm infants (*n* = 106). Both UP and UMA were higher in preterm infants than in full-term infants. Preterm infants had a 1.49-fold increase in UP (full-term vs. preterm: 710.21 mg/g creatinine vs. 1056.87 mg/g creatinine). Preterm infants had a 1.47-fold increase in UMA (full-term vs. preterm: 7.30 mg/l vs. 10.73 mg/l). The dotted line indicates the normal range reference of UP and UMA in adults and children. (**C**, **D**) Both UP (*ρ* =  − 0.612, *p* < 0.001) and UMA (*ρ* =  − 0.255, *p* = 0.002) were negatively correlated with GA at birth. (**E**, **F**) Preterm infants were divided into three groups according to GA: 35–36 weeks (*n* = 27), 31–34 weeks (*n* = 38), and ≤ 30 weeks (*n* = 41). All three preterm groups demonstrated significantly increased UP and UMA levels. The level of UP in the ≤ 30-week group was higher than that in the 35–36-week group (*p* = 0.011). No difference was noted among other preterm groups. For UMA, no difference was found among all preterm groups (Mann–Whitney rank sum test was used to compare UP and UMA between preterm and full-term infants, *p < 0.05)

In addition to urine podocyte detection, UP and UMA levels were also detected. As shown in Fig. 3A and B, although UP and UMA levels of nearly all preterm infants were considered in the normal range (dotted line), significant differences in UP (*Z* =  − 0.6966, *p* < 0.001) and UMA (*Z* =  − 0.4324, *p* < 0.001) were found between the preterm group and full-term group. We also analyzed the correlation between UP and UMA and GA, and the results showed that both UP (*ρ* =  − 0.612, *p* < 0.001) and UMA (*ρ* =  − 0.255, *p* = 0.002) were negatively correlated with GA, and the smaller the GA, the higher the UP and UMA levels. Figure 3E and F show that the levels of UP and UMA were increased in all three preterm groups.

### Podocyte loss in relation to proteinuria and microalbuminuria

**Fig. 4:**
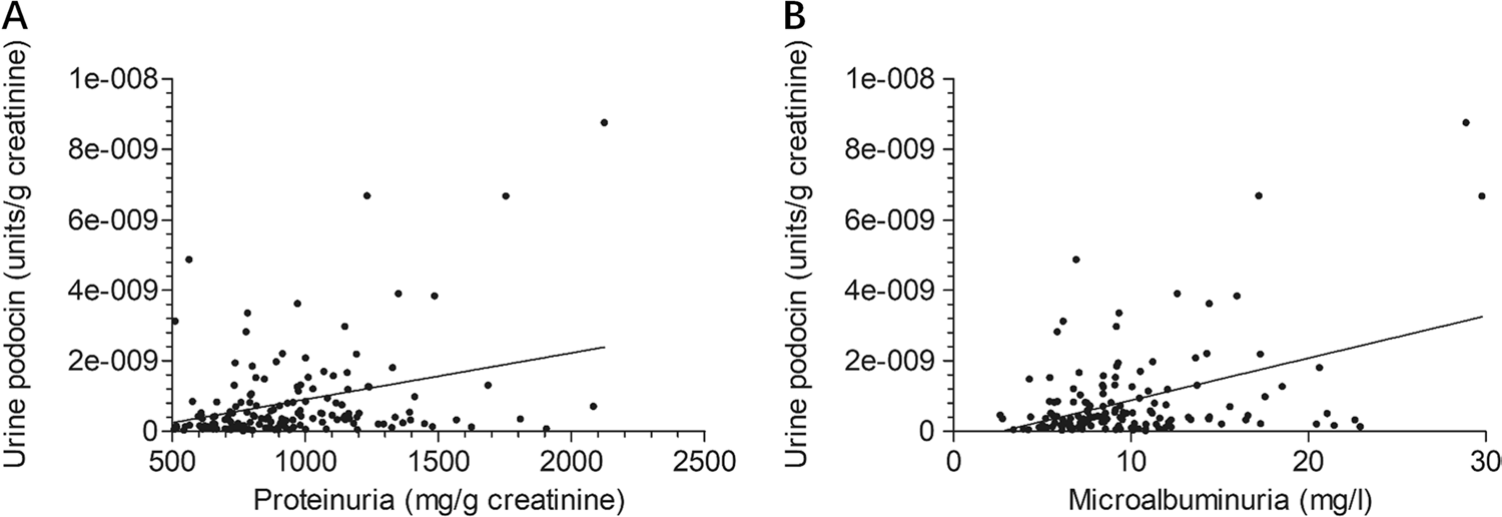
Urine podocyte mRNA level in relation to UP and UMA. Spearman rank correlation test was performed to examine the correlation between urine podocyte mRNA and UP (**A**) as well as urine UMA (**B**), and positive correlations were found between urine podocyte loss and UP (*ρ* = 0.268, *p* = 0.001) as well as UMA (*ρ* = 0.263, *p* = 0.001)

Figure 4A and B show that UpodCR was positively correlated with either proteinuria (*ρ* = 0.268, *p* = 0.001) or microalbuminuria (*ρ* = 0.263, *p* = 0.001)

### Influence of perinatal factors on podocyte loss

Preterm infants are often exposed to various perinatal factors. In addition to GA, we also analyzed the effects of various perinatal factors on UpodCR, which indicates podocyte loss. The results of the univariate regression analysis of each perinatal factor and podocyte loss are shown in Table 2. GA, birth weight, birth length, Apgar score at 1 min, singletons, retinopathy of prematurity, and infection were correlated with podocyte loss. For most preterm infants, the smaller the GA, the lighter the birth weight and the shorter the birth height. These three perinatal factors could be interpreted by GA. Moreover, in this study, the Apgar score at 1 min was strongly affected by GA. Thus, we included GA at birth, small for gestational age (SGA), singletons, maternal age, neonatal infection, and retinopathy of prematurity in the multivariate analysis, and the results showed that GA and SGA were high-risk factors for podocyte loss (Table 3). According to the data in Table 3, the relative risk of podocyte loss could also be presented; for example, preterm infants with a GA of 27 weeks would have 0.2*10^−9^ units/g creatinine additional increased urine podocyte mRNA depletion when compared to preterm infants with a GA of 28 weeks.

**Table 2:** Results of univariate linear regression analysis of perinatal risk factors and urine podocyte loss

Variable	Range	*β* (95% CI)	*p*
Gestation (weeks)	24–42	** − 0.055 (− 0.074; − 0.036)**	** < 0.001**
Sex	1 = male 2 = female	0.115 (**− **0.062; 0.291)	0.201
Delivery mode	1 = vaginal delivery 2 = cesarean delivery	** − **0.086 (**− **0.263; 0.090)	0.334
Apgar score (at 1 min)	1–10	** − 0.081 (− 0.144; − 0.019)**	**0.011**
Single birth or not	1 = single birth 2 = multiple births	**0.232 (0.050; 0.414)**	**0.013**
Birth weight (g)	860–3960	**0.000 (0.000; 0.000)**	** < 0.001**
Birth height (cm)	34–53	** − 0.041 (− 0.057; − 0.026)**	** < 0.001**
Small for gestational age	1 = yes 2 = no	** − **0.230 (**− **0.462; 0.001)	0.051
Maternal age (years)	18–45	** − **0.015 (**− **0.036; 0.005)	0.142
Maternal diabetes mellitus	1 = yes 2 = no	** − **0.094 (**− **0.315; 0.127)	0.400
Maternal hypertension	1 = yes 2 = no	** − **0.028 (**− **0.275; 0.219)	0.822
Preeclampsia	1 = yes 2 = no	0.028 (**− **0.263; 0.319)	0.850
Neonatal intracranial hemorrhage	1 = yes 2 = no	** − **0.083 (**− **0.383; 0.217)	0.585
Retinopathy of prematurity	1 = yes 2 = no	** − 0.493 (− 0.833; − 0.153)**	**0.005**
Neonatal infection	1 = yes 2 = no	** − 0.251 (− 0.432; − 0.070)**	**0.007**
Administration of ibuprofen	1 = yes 2 = no	** − **0.133 (**− **0.379; 0.113)	0.288

Bold: Variable is statistically correlated with podocyte loss with *p* < 0.05

**Table 3:** Results of multiple regression analysis of perinatal risk factors and urine podocyte loss

Variable	Range	*β* (95% CI)	*p*
Constant	—	** − 7.007 (− 7.770; − 6.244)**	< 0.001
Gestation (weeks)	24–42	** − 0.056 (− 0.074; − 0.037)**	< 0.001
Small for gestational age	1 = yes; 2 = no	** − 0.263 (− 0.472; − 0.055)**	0.014

Bold: Variable is statistically correlated with podocyte loss with *p* < 0.05. In stepwise regression model, “single birth or not,” “retinopathy of prematurity,” and “neonatal infection” were all included as covariates in the first step, but these variables were removed from models step by step since all *p* > 0.05

## Discussion

In this study, podocyte loss in preterm infants and related perinatal risk factors were examined by detecting podocyte mRNA levels in the urine. The results revealed that preterm infants had a higher level of urine podocyte loss. Urine podocyte loss was also negatively related to GA and positively related to proteinuria and microalbuminuria. GA and SGA were also risk factors for podocyte loss.

In kidney diseases, urinary podocyte loss was often 10- to 100-fold higher in the disease group than in the control group. This level of podocyte loss in the disease group has already been proven to be the driving factor of proteinuria, glomerulosclerosis, further reduction of glomerular filtration rate, and CKD (podocyte depletion hypothesis) [20–23, 25, 26]. In the present study, only an approximately fivefold higher podocyte loss was detected in the preterm group than in the full-term group. This fivefold change in podocyte loss is mild when compared to that in kidney diseases. Whether such mild changes could account for a poor prognosis in the development and progression of CKD is still unknown. Nevertheless, in adults, Naik et al. deduced that a threefold increase in podocyte detachment may play a potential role in causing development of CKD and kidney failure in an indirect way [27]. However, the prerequisite for poor prognosis is persistent podocyte loss over time. In the present study, increased podocyte loss was limited to the neonatal period in preterm infants, and it is still unclear whether persistent podocyte loss would continue to childhood and adulthood. Although there were no follow-up data of podocyte loss in preterm infants, our animal data showed that accelerated podocyte loss was detected in preterm rats from the age of 3 weeks to the age of 12 months (corresponding to childhood to adulthood). Thus, if we assume that increased podocyte loss in preterm infants would persist during childhood and adulthood, similar to what we found in preterm rats, the initial fivefold increased podocyte loss caused by preterm birth could be speculated to be one of the risk factors for the future development of CKD.

In this study, podocyte loss was interpreted based on podocyte-specific podocin mRNA levels in the urine pellet. Podocyte-specific mRNA or podocyte-specific protein from the urine pellet has been widely used in several kidney studies to indicate podocyte loss from the kidney and have also been identified as a parameter preferable to cell number to indicate podocyte loss and podocyte injury [25–31]. Such as in Hara et al., podocyte-specific podocalyxin from urine pellets has been confirmed as an indicator for podocyte injury in nephrotic syndrome and nephritis [31]. Thus, in this study, podocyte loss was based on podocyte-specific podocin mRNA levels extracted from the urine pellet from infants.

UP and UMA have already been proven to be independent risk factors for CKD development [32–35] and these two factors were also measured and explored in relation to podocyte loss in the present study. First, a correlation between UP and UMA with GA was also detected in the present study (Fig. 3C and D). This result was similar to previous studies [36–41], and UP and UMA were inversely associated with GA at birth. Then, the relationships between UP, UMA, and podocyte loss were examined and positive correlations were found between them (Fig. 4). However, there is a weak correlation between UP and podocyte loss. The high level of UP in preterm infants may indicate structural immaturity, glomerular injury, or tubular injury and may represent a poor prognosis. The weak correlation between UP and podocyte loss suggested that prematurity not only causes podocyte injury but also some non-podocyte injury. Future studies should also consider non-podocyte kidney injury caused by preterm birth.

In addition, when comparing three preterm infant groups with full-term infants, not only UP and UMA (Fig. 3E and F) but also podocyte loss (Fig. 2B) were significantly higher than those of the full-term group (Figs. 2 and 3). Interestingly, in the 35–36 weeks preterm infant group, all three parameters, including UpodCR, UP, and UMA, were significantly higher than those of the full-term group (Figs. 2 and 3). This result suggested that there was a high risk of kidney injury also present in the 35–36 weeks preterm infant group. Previous studies have shown that this near-term preterm group has a high risk of other neonatal morbidities such as respiratory diseases, digestive diseases, and infectious diseases [42]. Thus, attention should also be paid to preterm infants born during this period.

In our animal study [19], a preterm rat model was established by delivery 2 days early. This preterm rat model could not completely simulate the conditions of preterm infants because these infants have experienced various adverse events, such as asphyxia, hypoxia, drug exposure, acute kidney injury, infection, and maternal influence [43, 44]. In addition, as shown in Fig. 2A, although GA was negatively correlated with podocyte loss, the relationship between them was weak. Some extremely preterm infants with GA less than 28 weeks presented low levels of podocyte loss while some near-term preterm infants showed high levels of podocyte loss, which suggested that GA may not be the only factor affecting podocyte loss; the relative perinatal factors may also have an important effect on podocyte loss. Several perinatal factors were correlated with podocyte loss (Table 2). The Apgar score at 1 min could reflect asphyxia and oxygenation at the same GA; previous studies have proven that a lower Apgar score predicts a poorer prognosis in neonates [45–48]. A correlation between the Apgar score at 1 min and podocyte loss may also indicate poor kidney function. However, this result may also be partially interpreted by GA because most cases with lower Apgar score at 1 min were also mainly related to extremely preterm infants. In the present study, no relationship was found between several maternal factors, such as gestational diabetes, pre-eclampsia, and podocyte loss. Regarding neonatal morbidity, retinopathy of prematurity and infection were correlated with podocyte loss. Retinopathy of prematurity could represent oxidation damage and oxidative stress in preterm infants [48, 49]. Several studies have reported that oxidative stress could aggregate podocyte injury in kidney diseases [50, 51]. Regarding infection in adults and children, several studies have already proven that infection could lead to podocyte injury and even acute kidney injury [23, 52, 53]. However, few studies have focused on oxidative stress or infection in preterm infants with kidney injury. This could be a potential research direction in this field. Moreover, SGA is often present in many chronic diseases related to preterm birth or low birth weight. This factor was strongly correlated with intrauterine growth restriction in infants or fetuses with fewer nephrons, which inhibited nephrogenesis [54, 55]. The low number of nephrons in preterm or low birth weight infants was hypothesized to be the major cause of future CKD development in recent years [14, 16, 18, 54, 55]. As shown in Table 3, SGA was identified as a risk factor for increased podocyte loss, which suggests that podocyte loss may also play a role in the development of future CKD in a cohort of SGA infants.

This study has some limitations. First, this study followed a cross-sectional design, which means that causality cannot be addressed. Second, podocyte loss was only examined in neonates. This study did not investigate whether a higher rate of podocyte loss in the preterm infant group would prevail until childhood and adulthood. In addition, although some perinatal risk factors were identified, there are still a small number of preterm infants distributed to each GA. Preterm infants with different GA are affected by different prenatal factors. Thus, more detailed information may not be recognized in the present study. Future studies could also focus on more detailed perinatal risk factors because they could be future intervention directions for relieving podocyte loss and then decreasing the risk of development of CKD.

In conclusion, the results of this study suggest high levels of podocyte loss in preterm infants. In addition, several perinatal factors were associated with podocyte loss and may be a potential direction for comprehensive research and intervention in this field.

## Supplementary Information

Below is the link to the electronic supplementary material.

Graphical abstract (PPTX 117 KB)
