# Supplementary material for: Urine podocyte mRNA loss in preterm infants and related perinatal risk factors
Source: Pediatr Nephrol. 2022 Jun 27;38(3):729–38. doi: 10.1007/s00467-022-05663-6 (PMC9842585; doi:10.1007/s00467-022-05663-6)
Supplement: Supplementary file 1 — Graphical abstract (PPTX 117 KB) [file 467_2022_5663_MOESM1_ESM.pptx]

## Slide 1
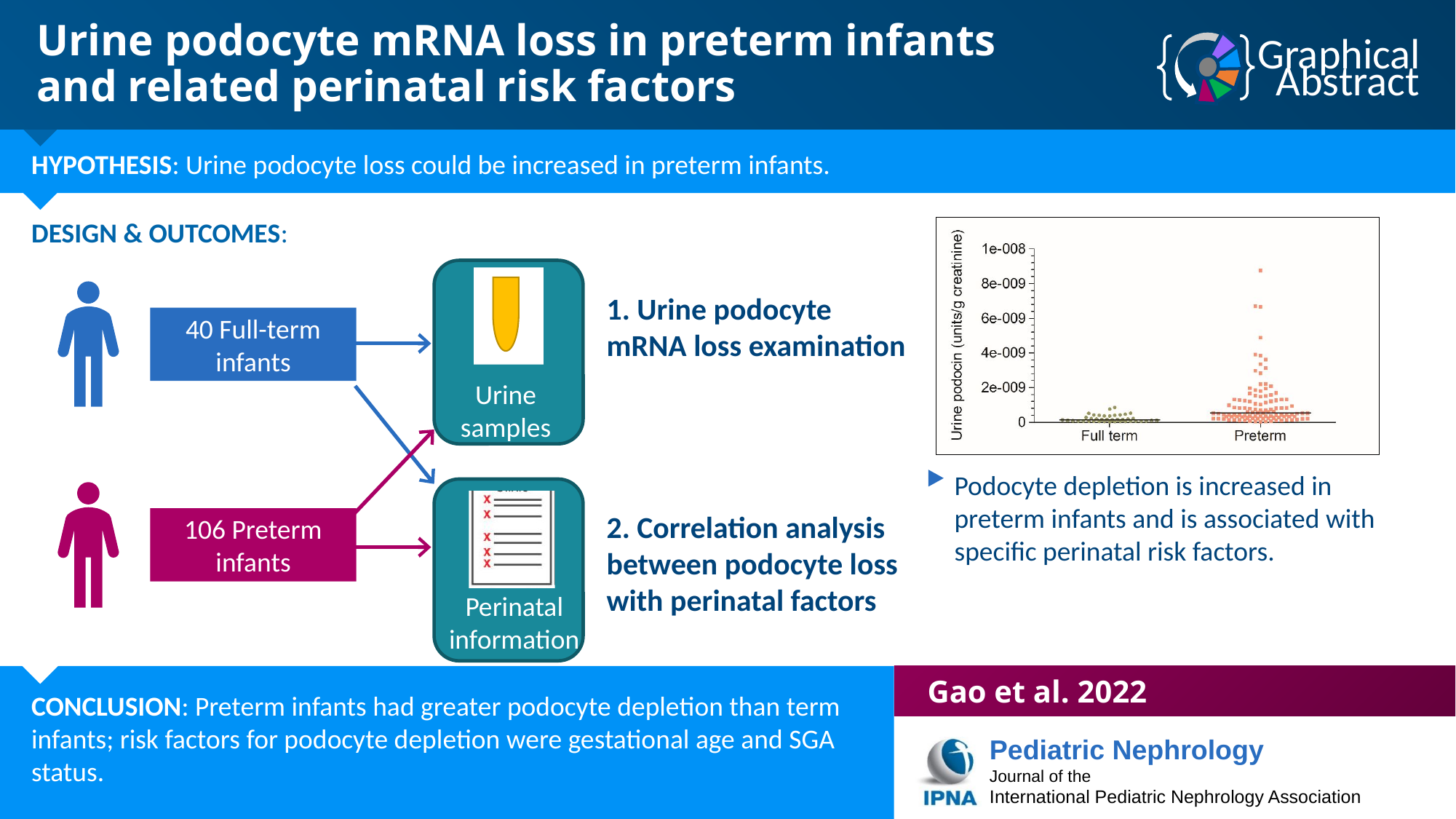

Urine podocyte mRNA loss in preterm infants
and related perinatal risk factors
HYPOTHESIS: Urine podocyte loss could be increased in preterm infants.
DESIGN & OUTCOMES:
Urine samples
1. Urine podocyte mRNA loss examination
2. Correlation analysis between podocyte loss with perinatal factors
40 Full-term infants
Podocyte depletion is increased in preterm infants and is associated with specific perinatal risk factors.
Perinatal information
106 Preterm infants
Gao et al. 2022
CONCLUSION: Preterm infants had greater podocyte depletion than term infants; risk factors for podocyte depletion were gestational age and SGA status.
